# Termination of pregnancy for fetal anomaly: a systematic review of the healthcare experiences and needs of parents

**DOI:** 10.1186/s12884-022-04770-4

**Published:** 2022-05-26

**Authors:** Suzanne Heaney, Mark Tomlinson, Áine Aventin

**Affiliations:** 1 School of Nursing and Midwifery, Queen’s University Belfast, 97 Lisburn Road, MBC Building, BT9 7BL Belfast, Northern Ireland; 2 Department of Global Health, Institute for Life Course Health Research, Stellenbosch University, Cape Town, South Africa

**Keywords:** Termination of pregnancy, Fetal anomaly, Congenital abnormalities, TOPFA, Abortion, Feticide, Healthcare experience, Healthcare needs, Parents, Systematic review

## Abstract

**Background:**

Improved technology and advances in clinical testing have resulted in increased detection rates of congenital anomalies during pregnancy, resulting in more parents being confronted with the possibility of terminating a pregnancy for this reason. There is a large body of research on the psychological experience and impact of terminating a pregnancy for fetal anomaly. However, there remains a lack of evidence on the holistic healthcare experience of parents in this situation. To develop a comprehensive understanding of the healthcare experiences and needs of parents, this systematic review sought to summarise and appraise the literature on parents’ experiences following a termination of pregnancy for fetal anomaly.

**Review question:**

What are the healthcare experiences and needs of parents who undergo a termination of pregnancy following an antenatal diagnosis of a fetal anomaly?

**Methods:**

A systematic review was undertaken with searches completed across six multi-disciplinary electronic databases (Medline, Embase, PsycINFO, CINAHL, Web of Science, and Cochrane). Eligible articles were qualitative, quantitative or mixed methods studies, published between January 2010 and August 2021, reporting the results of primary data on the healthcare experiences or healthcare needs in relation to termination of pregnancy for fetal anomaly for either, or both parents. Findings were synthesised using Thematic Analysis.

**Results:**

A total of 30 articles were selected for inclusion in this review of which 24 were qualitative, five quantitative and one mixed-methods. Five overarching themes emerged from the synthesis of findings: (1) Contextual impact on access to and perception of care, (2) Organisation of care, (3) Information to inform decision making, (4) Compassionate care, and (5) Partner experience.

**Conclusion:**

Compassionate healthcare professionals who provide non-judgemental and sensitive care can impact positively on parents’ satisfaction with the care they receive. A well organised and co-ordinated healthcare system is needed to provide an effective and high-quality service.

**Trial Registration:** PROSPERO registration number: CRD42020175970.

**Supplementary information:**

The online version contains supplementary material available at 10.1186/s12884-022-04770-4.

## Introduction

Congenital anomalies are defined as structural or functional abnormalities that occur prior to birth [1], often resulting in high levels of morbidity and mortality [2–5]. Improved technology and advances in clinical testing have resulted in increased detection rates of congenital anomalies during pregnancy [6–9]. This is mainly seen in high-income countries where sufficient resources and equipment enable increased routine antenatal testing and screening by adequately trained health professionals [10–14]. However, while detection rates have improved, few in-utero treatments are available for major anomalies leaving parents with limited options following diagnosis [15–17]. They can continue with the pregnancy, or in the case of the detection of a fetal anomaly, they can request a Termination of Pregnancy (TOP) in the 113 (57%) countries and territories around the world where it is legal to do so [9, 18-20]. Of the 86 countries and territories where TOP is prohibited, 81% are low- and middle-income countries [19].

In countries where TOP is legal there are often variations in the definition and clauses regarding the type of anomalies for which termination of pregnancy for fetal anomaly (TOPFA) is permitted. For example, in the United Kingdom (UK) the law stipulates that where the anomaly represents a ‘substantial risk’ that the child who would be born would be ‘seriously handicapped’, would be accepted as grounds (Ground E) for a TOPFA [21]. Conversely, in the Republic of Ireland two medical practitioners must be of the agreed opinion that the baby will die during pregnancy, labour or within twenty-eight days of birth for a TOPFA to be sanctioned [22]. In the United States, access to TOP varies from state to state, due to differing governments, healthcare providers and medical insurance restrictions [23]. Six states entirely prohibit TOPFA, while a further four require mandatory counselling on available perinatal hospice services before it can be performed [24].

Legal differences regarding the type and degree of severity of congenital anomalies considered severe enough to access TOPFA, mean that obtaining accurate data pertaining to TOPFA is challenging [25–27]. European figures for TOPFA suggest a prevalence rate of 4.6 per 1,000 births [28]. In the UK, over 70% of congenital anomalies are detected during pregnancy, and, of those, around 37% will result in a TOPFA [29]. Government figures for England and Wales reported that, in 2019, 3,183 TOPFAs were carried out, in the context of 640,370 livebirths and 2,522 stillbirths in the same year [30, 31]. However, groups working in this area of healthcare, such as the British Pregnancy Advisory Service and Antenatal Results and Choices suggest that actual rates of TOPFA are higher, estimating a rate of 5,000 per year [32]. A possible reason for the disparity in figures could be that in the UK if a TOPFA is carried out under 24 weeks it could be recorded under Ground C of the Abortion Act (the risk to a woman’s physical or mental health is greater than if she continued the pregnancy), rather than Ground E [21]. Figures from 2019 state 98% of all abortions in England and Wales were performed under Ground C [30].

Existing reviews have explored the psychological experience and impact of TOPFA [33–38] yet there is limited consideration of the holistic healthcare experience of parents. One exception is Lafarge et al.’s study which offers a more holistic view and interpretation of the experience of TOPFA through a meta-ethnography of women’s experiences [39]. This study was not, however, specifically focused on healthcare experiences and did not include partners, an issue that is seen across the field of perinatal loss [40–43]. Another qualitative meta-synthesis review explores the labour and birth experiences of women who have had a TOPFA [44] and while offering useful insights, it only covers one part of the overall healthcare experience. Another review of the needs of women who have experienced a TOPFA did report needs related to the healthcare system, however, unlike the current review, it did not use systematic review methodology [45].

The dearth of research looking at the entire TOPFA healthcare experience, as well as from both parents’ experience warrants further investigation. Thus, the overall aims of this systematic literature review were to (a) synthesise findings from the international literature on the healthcare experiences and needs of parents who undergo a termination of pregnancy following an antenatal diagnosis of a fetal anomaly, (b) carry out a thematic analysis of the evidence, and (c) provide a comprehensive narrative synthesis, focusing on the views, experiences, feelings, opinions and needs of both parents.

## Methods

This mixed-methods systematic review adhered to a PROSPERO pre-registered protocol (CRD42020175970) [46]. It was conducted using Covidence Systematic Review Software [47], NVivo Version 12 for Windows [48] and in accordance with Preferred Reporting Items for Systematic Reviews and Meta-Analyses (PRISMA) 2020 guidelines [49].

### Search strategy and sources

The search strategy was developed by reviewing and extracting search terms from existing relevant reviews [39, 44] or published studies with a similar sample [50–52]. Six multi-disciplinary electronic databases (Medline, Embase, PsycINFO, CINAHL, Web of Science, and Cochrane) were searched individually on 6^th^ August 2021 using the search terms in Table 1. Manual searches were also conducted, and included: grey literature databases (Open Grey, BASE, GreyNet); clinical trial registers (Clinicaltrials.gov, ISRCTN Registry, NIHR UK, WHO ICTRP); web searches (Google, Google Scholar, Grey Literature Report, National Health Service (NHS) Evidence); dissertation/thesis searches (OATD International, ProQuest); and through examination of the reference lists of included studies. The lead author, SH carried out all searches.

**Table 1:** PICO Framework search terms

PICO Acronym	MeSH search terms	Additional search terms
Population	Parents, Parenting, Pregnant Women, Men, Postpartum Period, Peripartum Period, Women’s Health, Men’s Health, Maternal–Fetal Relations	Famil*, Parent*, Mother, Father, Wom?n, M?n, M?m, Dad, Maternal, Paternal, Pregnant Wom?n, Pregnant Person, Pregnan*
Intervention	Aborted Fetus, Abortion Applicants, Abortion, Eugenic, Abortion, Induced	Abortion, Termination, Fet?cide, Medical Abortion, Medical Miscarriage, Medical Termination, Induced Abortion, Termination of Pregnancy, TOP
Condition	Fetal Viability, Congenital Abnormalities, Congenital, Hereditary, and Neonatal Diseases and Abnormalities	F?etal Anomal*, Fatal F?etal Anomal*, Incompatible with life, Abnormality, Anomal* Scan, Life Limiting, Fatal Anomal*, Gene? Condition, Gene? Disorder, Congenital Anomal*, Congenital malformation, FA, FFA
Outcome		Experience*, Opinion*, View*, Need*, Health?, Healthcare, Health service, Support, Care, Access*, Travel*, Financ*, Cost, Stigma, Psychological outcome*, Physical outcome*, Patient satisfaction, Social support, Mental health, Family, Family relations, Family conflict, Maternal behavio?r, Paternal behavio?r, Life Change Events, Trauma?, Stress disorders, Life stress event$.tw, Health?related quality of life.tw, Parent morbidity.tw, Satisfaction with care.tw

### Study selection

Included studies were of qualitative, quantitative or mixed methods designs reporting the results of primary data on the healthcare experiences or healthcare needs in relation to TOPFA for either or both parents. Due to time and resource constraints a ten-year timeframe was implemented, with searches limited to studies published between 1^st^ January 2010 and 6^th^ August 2021. No limitation was set on the type of anomaly or study location. Exclusion criteria included: studies not published in English; non-empirical studies, such as case reports, opinion pieces or reviews; and studies reporting experience of TOP for a reason other than fetal anomaly. Studies were also excluded if they only reported health professionals’ or other family members’ experiences of TOPFA.

### Search terms

A combination of Medical Subject Headings (MeSH) and keyword search terms were employed, following consultation with a search specialist. Search terms used were structured within the PICO framework (see Table 1) [53].

SH screened all articles and ÁA independently screened a randomly selected 10% (*n* = 9) of the full text articles for eligibility.

### Quality appraisal

While acknowledging the lack of consensus relating to how, what (and perhaps if) qualitative research should be quality assessed [54–56], we carried out quality assessment for the purposes of rigour and transparency [57]. The Mixed Methods Appraisal Tool (MMAT) Version 2018 [58] was used given its value for appraising the quality of a variety of study designs [59]. The MMAT includes a total of 25 criteria and two screening questions. It can appraise five categories of study design: (1) qualitative, (2) randomised controlled, (3) non-randomised, (4) quantitative descriptive and (5) mixed methods. The screening questions help to exclude non-empirical studies. For each study design, there are five core criteria to appraise the methodological quality of studies [see 58]. Each criterion is rated as ‘yes’, ‘no’ or ‘can’t tell’.

The quality of studies was evaluated primarily on the appropriateness of the study design and interpretation of the results substantiated by findings and data. As per the guidelines of the tool no overall score was calculated. In line with MMAT guidelines, two reviewers (SH and ÁA) were independently involved in the appraisal process [58]. SH reviewed all of the included studies and ÁA assessed the quality of a random sample of the articles (10%). Agreement about the articles to include in the review was high. Any divergence of opinion was resolved through discussion.

### Data extraction and synthesis

Descriptive data were initially extracted by SH into the Covidence Systematic Review Software [47], using a pre-designed data extraction form to collate and manage the information. ÁA cross-checked this information. The descriptive data extracted summarised the key characteristics of the selected studies and included: year of publication, the country where the research was conducted, study design, participants, data collection methods and analysis, study aims, and main findings.

Next, empirical findings were imported to NVivo Version 12 for Windows [48]. The findings were then thematically synthesised [57] allowing for the analysis of both qualitative and quantitative data regarding experiences and perspectives within a healthcare context [60]. This inductive approach comprised three stages: 1) line-by-line coding; 2) organisation of codes into descriptive themes; and 3) development of analytical themes [61, 62]. To foster rigour of the coding process, ÁA independently coded a randomly selected 10% of the included articles [63]. This was followed by regular discussions amongst authors to examine emerging codes, connections, meanings and themes. Consensus between the reviewers remained high throughout the process.

## Results

**Fig. 1:**
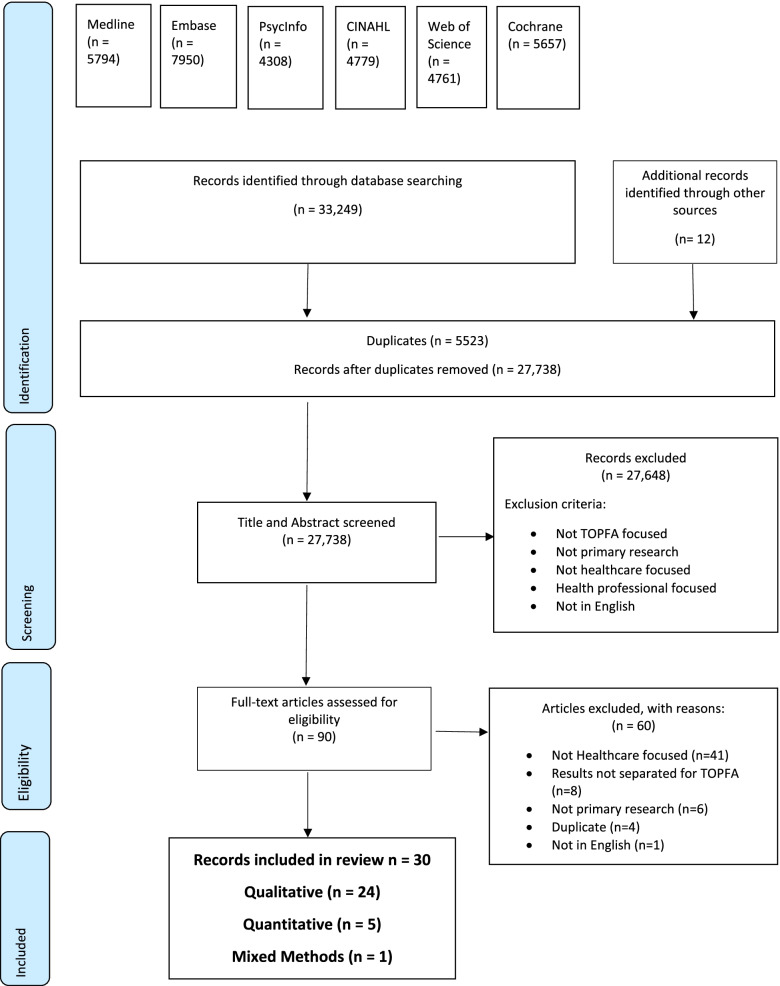
Search strategy and identification of articles included in this review

A total of 33,249 articles were identified through the six databases used in this review. A further 12 articles were found as a result of the additional searches. A PRISMA flowchart (Fig. 1) presents an overview of the identification and screening process of included studies. 5,523 duplicates were removed, the title and abstract of the remaining 27,738 articles were screened and 90 articles were assessed as eligible for full text screening. The full text of 90 articles were screened, 60 of which were excluded. A total of 30 articles were selected for inclusion in this review and were agreed upon by all authors.

### Overview of included studies

Of the thirty articles, 24 were of a qualitative nature, five quantitative and one mixed-methods [64–93]. The findings presented are based on twenty-eight studies as findings from two studies are reported in four articles [71–72; and 76–77]. Study characteristics are summarised in Table 2.

**Table 2:** Characteristics of included studies

Study	Country	Participants	Data Collection	Methodology	Study Aim	Main Findings
Asplin et al., 2014 [64] **Pregnancy termination due to fetal anomaly: Women’s reactions, satisfaction and experiences of care**	Sweden	11 women	Semi-structured interviews	Qualitative Exploratory Descriptive Design Retrospective	The aim of the study was to explore what women who have had a pregnancy termination due to a detected fetal malformation perceive as being important in their encounters with caregivers for promoting their healthy adjustment and well-being	The most important factors associated with satisfaction regarding pregnancy termination due to a fetal malformation are the human aspects of care Main theme: State-dependent communication and in-depth understanding and compassion Categories and sub-categories: 1. Satisfaction with care 2. Management of feelings and reactions Communication and how to be acknowledged and thus experience support 3. Structure and information 4. In-depth understanding and compassion 5. Sadness and frustration as reactions and part of adaption 6. Follow up care
Atienza-Carrasco et al., 2020 [65] **Experiences and outcomes following diagnosis of congenital foetal anomaly and and medical termination of pregnancy: A phenomenological study**	Spain	27 women	Nonparticipant observation Semi-structured interviews	Qualitative Phenomenological Approach Retrospective	To determine and describe the experiences of pregnant women who receive a diagnosis of chromosomopathy and/or foetal malformation during a prenatal check-up and who decide to legally terminate the pregnancy	Themes and subthemes 1. Communication of the diagnosis (clinical relationship; environment; how was the diagnosis reported?) 2. Emotional impact (reactions provoked by the news; treatment possibilities; time allowed for reflection; social representations of motherhood) 3. Termination of the pregnancy (decision-making; reasons for terminating the pregnancy; what was the patients’ experience?; grief) 4. Psychosocial support (perceptions of the help and support received; future expectations)
Carlsson et al., 2016 [66] **Experiences of termination of pregnancy for fetal anomaly: A qualitative study of virtual community messages**	Sweden	112 women 1 man 9 not disclosed	Cross-sectional study of messages in virtual communities	Qualitative Content analysis Retrospective	To explore experiences described by posters in Swedish virtual communities before, during and after termination of pregnancy due to a fetal anomaly	Before termination of pregnancy: 1.E1. Emotional shock 2.D2. Difficult decision During termination of pregnancy: 1. Compassionate care from present caregivers 2. Emotional and physical pain 3. Lack of understanding about termination of pregnancy 4. Viewing the fetus After the termination of pregnancy: 1. Coming to terms with the decision 2. Perinatal loss 3. Fears of recurrence 4. Longing for a child
Chaloumsuk, 2013 [67] **Women’s experiences of miscarriage and termination of pregnancy for fetal anomaly in Thailand: A phenomenological study**	Thailand	12 women	Unstructured interviews	Qualitative Interpretive phenomenology Retrospective	This study aimed to gain an understanding of experiences of miscarriage and termination of pregnancy for fetal anomaly among a group of Thai women	Main themes: 1. Facing the loss of hope 2. Gaining emotional balance 3. Need for intervention
Cowchock et al., 2011 [68] **Spiritual needs of couples facing pregnancy termination because of fetal anomalies**	USA	9 women 5 men	Survey	Quantitative Survey Retrospective Measures Used: *“In this pilot study we adapted some questions from a published scale developed by the Healthcare Chaplaincy in New York, NY. (Flannelly KJ *et al*., 2006) We asked these couples which members of the health care team should address their spiritual concerns, and whether attention to these needs would improve their satisfaction with their obstetric care. Because no similar published survey was available from this population as a guide, we added an exploratory qualitative component: Three short narrative questions asked for examples of the “best” and “worst” incidents surrounding this loss and the parents’ own statement of what responses would be most helpful.”*	The spiritual needs of couples (9 mothers and 5 fathers) who were planning to terminate wanted second trimester pregnancies because of serious fetal anomalies were surveyed	Couples’ greatest needs were for a “guidance from a higher power” and for “someone to pray for them.” Parents did not want or expect their healthcare team to discuss their faith, or to pray with them. Most would prefer support from their own pastors. Their religious community was involved to only a small extent. Parents would welcome support from hospital chaplains, who could play a substantive and unique pastoral role in this clinical context
Dekkers et al., 2019 [69] **Termination of pregnancy for fetal anomalies: Parents’ preferences for psychosocial care**	The Netherlands	76 women 36 partners	Semi-structured online questionnaire	Quantitative Cross-sectional Retrospective cohort study Retrospective Measures Used: *“The authors developed a semi‐structured online questionnaire based on a questionnaire used by Levert *et al*. (2017) which aimed to study the psychosocial care (PSC) needs of children with coronary heart disease and their parents. Adjustments were made as necessary for the specific needs of the respondents in this study. These adjustments were derived from the international literature (Ramdaney *et al*., 2015; Lafarge *et al*., 2014) and from the clinical expertise of the researchers* *The questionnaire assessed whether women and partners would have liked to receive PSC on a variety of issues. It consisted of 90 multiple‐choice questions and 12 open‐ended (not mandatory) questions.”*	To investigate, from the perspective of women and partners, at what stage of a termination of pregnancy for fetal anomalies psychosocial care (PSC) is most meaningful, what topics should be discussed, and who should provide PSC	Overall, women expressed a greater need for PSC than their partners. Parents expressed a preference for receiving support from a maternal‐fetal medicine specialist to help them understand the severity and consequences of the anomalies found and to counsel them in their decision regarding termination. Parents showed a preference for support from mental healthcare providers to help with their emotional responses. Forty‐one percent of the women visited a psychosocial professional outside of the hospital after the TOP, indicating a clear need for a well‐organised aftercare
Desrochers, 2011 [70] **The psychosocial impact of termination of pregnancy for fetal anomaly on the male partner**	USA	7 men	Semi-structured interviews	Qualitative Retrospective	The purpose of this study was to explore the thoughts and feelings of fathers throughout the TOPFA experience including the initial diagnosis and the decision-making process, as well as the journey of grieving and coping	Themes and subthemes: 1. Shock and devastation 2. Discomfort with options 3. Most emotional time (making the decision; viability not associated with difficulty of decision) 4. External loss issues (society) (Telling other people/family; difficult to see other pregnant women/babies) 5. Internal loss issues (family) (partner’s grief response; strong effect on relationship) 6. Diversity in coping styles 7. Internalize feelings 8. Importance of support (genetic counselors; others)
Fisher and Lafarge, 2015 [71] **Women’s experience of care when undergoing termination of pregnancy for fetal anomaly in England**	UK (England)	361 women	Cross sectional online survey	Qualitative Retrospective	This study investigated women’s experience of care when undergoing termination of pregnancy for fetal anomaly	Five themes were identified as underpinning what women considered ‘good care’: 1. Being cared for in a timeframe and environment that feels right 2. Receiving the right level of care 3. The role of healthcare professionals and support organisations 4. Acknowledging women’s particular circumstances 5. Enabling women to make choices
Fisher et al., 2015 [72] **Termination for fetal anomaly: Are women in England given a choice of method?**	UK (England)	351 women	Self-administered online questionnaire	Quantitative Retrospective Measures Used: An anonymous, self-administered online questionnaire, which was developed with the help from the Expert Advisory Group. The aim of the questionnaire was to determine if women undergoing TFA in England were offered a choice of method and what factors influenced the offer of a choice. Information on women's experiences of TFA was also collected	The authors investigated whether women are offered a choice of method, by surveying members of a UK parent support organisation (Antenatal Results and Choices)	Main findings: 1. Mean gestational age at TOPFA was 17 weeks 2. 14% (*n* = 50) were offered a choice of method, falling to 8% (*n* = 19) after 14 weeks' gestation 3. Overall, 78% (*n* = 275) underwent medical TOPFA with 88% stating they chose it because it was the only method offered 4. 60% (*n* = 30) of those offered a choice had a surgical TFA 5. The survey suggests that women having TFA are not offered a choice of method
Gawron et al., 2013 [73] **An exploration of women’s reasons for termination timing in the setting of fetal abnormalities**	USA	30 women	Semi-structured interviews	Qualitative Latent content analysis Retrospective	The objectives of this study were to explore reasons for pregnancy termination timing among patients with fetal abnormalities by analyzing their pregnancy care timeline and their decision-making process	Main themes: 1. An abrupt shift in “low-risk” pregnancy perception 2. Challenging medical interactions 3. An emotional decision-making process 4. Termination access barriers
Hassan, 2015 [74] **Women’s long term life experience after pregnancy termination for fetal abnormality: Interpretive phenomenological study**	Canada & USA	10 women	Un-structured in-depth interviews	Qualitative Interpretive phenomenology Retrospective	To gain an in-depth understanding of the long-term experiences of women who terminated their pregnancy for fetal abnormalities and reveal the meanings embedded in their experiences	Six themes were identified as characteristics of the women's experiences over time: Encountering the unexpected 1. Making sense of the unexpected 2. Facing the inevitable decision 3. Living with the decision 4. Feeling supported 5. Changing perspectives
Irani et al., 2019 [75] **Emotional and cognitive experiences of pregnant women following prenatal diagnosis of fetal anomalies: A qualitative study in Iran**	Iran	7 women	Semi-structured in-depth interviews	Qualitative Conventional content analysis Retrospective	To explore the emotional and cognitive experiences of pregnant women following prenatal diagnosis of fetal anomalies in Mashhad, Iran	Four categories and 10 subcategories emerged: 1. Category one—grief reactions during the time of diagnosis (shocked and panicked; distressed and disbelieved) 2. Category two—perinatal loss through a pregnancy termination (guilt and shame during pregnancy termination; loss of their expected child; suffering and emotional distress process; unmet needs by health professionals) 1. Category three—fears of recurrence in future pregnancies (worried about inadequate prenatal care in the future pregnancies; worried about abnormal fetus in next pregnancies) 2. Category four—a dilemma between hope and worries (hope for normality; worried about future)
Kamranpour et al., 2020 [76] **A qualitative study exploring the needs related to the health system in women with experience of pregnancy termination due to fetal anomalies in Iran**	Iran	25 women 2 men	In depth semi-structured interviews	Qualitative Conventional qualitative content analysis Retrospective	This study aimed to explore the needs related to the health system in women with experience of pregnancy termination due to fetal anomalies	Needs related to the health system in women with experience of pregnancy termination due to fetal anomalies were categorized in the three main categories: 1. Efficient treatment team 2. Optimal organizational structure in providing services 3. Financial support for families”
Kamranpour et al., 2021 [77] **Termination of pregnancy for fetal anomalies: a qualitative study of the informational and educational needs of women**	Iran	25 women 2 men	In depth semi-structured interviews	Qualitative Conventional qualitative content analysis Retrospective	This study aimed to explore the informational and educational needs of women who have experienced pregnancy termination because of fetal anomalies	The informational and educational needs of women who have experienced pregnancy termination because of fetal anomalies were categorized in three main categories: 1. Receiving information tailored to the client's circumstances 2. Learning life skills to cope 3. Getting prepared for the next pregnancy
Kecir et al., 2020 [78] **Experiences of fathers having faced with termination of pregnancy for foetal abnormality**	France	8 men	Semi-structured interviews	Qualitative Retrospective	The aim of this qualitative study was to describe how fathers perceive the TOPFA, their feelings about caregivers and their strategies for coping	Main themes: 1. Reaction to diagnosis and decision making 2. Emotional dimension 3. Perception of his place (father) next to his spouse 4. Adaptation strategies 5. Experience of support rituals 6. Expectations and reservations regarding the care system 7. Expectations with regard to society
Koponen et al., 2013 [79] **Parental and professional agency in termination for fetal anomalies: analysis of Finnish women’s accounts**	Finland	8 women	Written accounts	Qualitative Linguistic discourse analysis Retrospective	This study explores the construction of parental and professional agency in the written accounts by women who have undergone selective abortion	The accounts indicate that the mothers themselves exhibited both strong and weak agency during the process of prenatal diagnosis. The role of the professionals was usually discussed in these accounts concerning only the phases of pregnancy when something out of ordinary had been detected. After the termination, the mothers expressed that they were forced to exhibit strong agency and find ways to cope with their distress unaided due to a lack of professional support
Lafarge et al., 2013 [80] **Women’s experiences of coping with pregnancy termination for fetal abnormality**	UK (England)	27 women	Online survey	Qualitative Interpretative phenomenological analysis Retrospective	To examine the coping strategies women use both during and after a TOPFA procedure	Coping with the procedure: 1. Rreceiving/giving support 2. Acknowledging the baby 3. Problem solving 4. Dissociating oneself from the procedure 5. Attributing meaning to the birth experience Post-termination coping: 1. Remembering the baby 2. Receiving/providing emotional support 3. Avoidance 4. Looking to the future
Lafarge et al., 2019 [81] **Pregnancy termination for fetal abnormality: Ambivalence at the heart of women’s experience**	UK (England) and France	27 women (England) 17 women (France) *UK sample is the same as study 80	Online survey (open-ended questions) Interviews	Qualitative Thematic analysis Retrospective	The aim of this article was to demonstrate the relevance of ambivalence to the experience of TOPFA. Data from two qualitative studies conducted with women who had undergone TOPFA, one in England, the other in France, was used to convey and illustrate the ambivalence that characterizes the TOPFA experience	The findings point to eight manifestations of ambivalence throughout the process of TOPFA: 1. Hope and despair 2. A choice but no choice 3. Standing still and rushing 4. Bonding and detaching 5. Trauma and peace 6. Disclosure and secrecy 7. Bridging past and future 8. Individual and societal experience
Leichtentritt, 2011 [82] **Silenced voices: Israeli mothers’ experience of feticide**	Israel	13 women	Semi-structured in-depth interviews	Qualitative Narrative analysis Retrospective	The study aimed to understand the experience of women who undergo feticide in Israel	Three main themes: 1. Difficult decision making process and outcomes 2. The unbearable experience of feticide 3. Feticide as an unspoken experience
Leichtentritt and Weinberg-Kurnik, 2016 [83] **No one sees the fathers: Israeli fathers’ experience of feticide**	Israel	17 men	Semi-structured in-depth interviews	Qualitative Hermeneutic phenomenology Retrospective	To examine the experience of Israeli fathers whose fetuses underwent feticide	The results indicate that men’s experiences in this arena are socially constructed and limited by gender roles and expectations Main themes: 1. The lack of a socially constructed terminology 2. Defining feticide: is it a loss? 3. Maintaining a masculine role: protecting self and others 4.”No man’s land”: exclusion and self-exclusion 5. Comprehensive understanding: counterfactual thinking
Leichtentritt and Mahat-Shamir, 2017 [84] **Mothers’ continuing bond with the baby: The case of feticide**	Israel	28 women	In-depth interviews	Qualitative Hermeneutic methodology Retrospective	The goal of this research was to reach an interpretive understanding of the continuing bond experience among Israeli mothers who underwent feticide, examining the strategies they use in maintaining a post death relationship with a child they did not know, whose death they chose and witnessed, within a social context that ignores their loss and forces them to silence their grief	The results highlight two themes: 1. Strategies for relinquishing connection with the baby 2. Strategies for maintaining a post-death relationship
Lotto et al., 2016 [85] **Care provision during termination of pregnancy following diagnosis of a severe congenital anomaly – A qualitative study of what is important to parents**	UK	10 women 8 men	Semi-structured interviews	Qualitative Constant comparative-based approach Retrospective	To understand the experiences of women and their partners following the decision to terminate a pregnancy affected by a severe congenital anomaly	The over-arching theme emerging from the data was that of 'falling through the gap', where the care received did not adequately meet the needs of women and their partners s1. Enacting the decision (consent; taking the tablets) 2. Labour and Birth (isolation; specialized facilities) 3. Moving on (seeing the anomaly; staying mum: disclosure and stigma; supporting the father)
Mitchell, 2016 [86] **“Time with babe”: seeing fetal remains after pregnancy termination for impairment**	Canada	19 women	Interviews	Qualitative Retrospective	To explore women’s responses to the opportunity to see their fetal bodies	1. Fetal visibility 2. Parental desire
Obst et al., 2021 [87] **Men’s experiences and need for targeted support after termination of pregnancy for foetal anomaly: A qualitative study**	Australia	10 men	Semi-structured interviews	Qualitative Thematic analysis Retrospective	This study aimed to explore men’s experiences of grief and support following TOPFA including how healthcare providers, systems and policies can best support men and their families	Thematic analysis resulted in the identification of three over-arching themes, each with two sub-themes 1. The most difficult choice (challenges of decision-making; stigma surrounding TOPFA) 2. Neither patient, nor visitor (Where do men fit?; dual need to support and be supported) 3. Meet me where I am (contact men directly; tailor support and services)
Pitt et al., 2016 [88] **Embodied experiences of prenatal diagnosis of fetal abnormality and pregnancy termination**	Australia	59 women	In-depth interviews	Qualitative Thematic analysis Retrospective	The aim of this study was to explore women’s embodied experiences of TOPFA	Two themes about embodiment were generated: 1. Transitioning embodiment (in-between embodiment; beginning the end; separating) 2. Vulnerable bodies in un/comfortable spaces (out of place in clinics and hospitals; home as place of sanctuary, isolation and remembrance)
Qin et al., 2019 [89] **Cognition, emotion, and behaviour in women undergoing pregnancy termination for foetal anomaly: A grounded theory analysis**	China	41 women	In-depth interviews	Qualitative Grounded theory Prospective	To understand the cognition, emotions, and behaviour of women who had recently undergone termination due to a foetal anomaly. In this study, the authors developed and tested a theoretical model to describe how women went through the process after termination	This study developed a cognitive-behavioural experience framework of women undergoing pregnancy termination due to a foetal anomaly. The model included 4 phases: 1. Denial Phase 2. Confirmation Phase 3. Decision-making Phase 4. Recovery Phase
Ramdaney et al., 2015 [90] **Support desired by women following termination of pregnancy for a fetal anomaly**	USA	51 women	Survey	Quantitative Prospective Measures Used: A 25-question survey, which included five questions on demographic information, five questions on pregnancy history, and 13 questions relating to the current pregnancy and support resources	The aim of this study was to identify what support, if any, women desire following a termination of pregnancy for a fetal anomaly	The authors studied the awareness and utilization of support resources in 51 women at the time of the procedure, at 6 weeks, and at 3 months following the event At the time of procedure: 1. 50% admitted contemplating their individualized need for support 2. Most expected to rely on the support of family and friends 3. 50% expressed the desire to commemorate the pregnancy 4. None wanted direct contact with their healthcare provider(s) Responses from the 6 weeks and 3 months assessments: 1. Many women indicated not coping as expected and were unprepared for the psychological consequences following the procedure 2. Findings indicated that women in these situations may not realize what their long-term support needs will be 3. Guidelines for routine follow-up care should be established among healthcare providers that respect this population's initial desires to avoid reminders of the pregnancy and promote a flexible timeframe for support uptake 4. Additional support resources that promote flexible uptake as well as meet the desires of anonymity and ease of access need to be developed for this population
Smith et al., 2020 [91] **The impact of genetic counselling on women’s grief and coping following termination of pregnancy for fetal anomaly**	USA	124 women	Online survey	Mixed-Methods Quantitative – both surveys were scored using respective analysis tools Qualitative – inductive content analysis Retrospective Measures Used: An online survey which included the brief COPE and the short version of the Perinatal Grief Scale	This study aimed to assess whether there is a significant difference in ability to cope post-procedure between women who see a genetic counselor and women who do not see a genetic counsellor prior to their termination for fetal anomaly	Main findings: 1. Women who saw a genetic counsellor (GC) utilized active coping, planning, and positive reframing significantly more than women who did not see a GC (*p* = 0.001, *p* = 0.031, *p* = 0.027, respectively) 2. GCs were perceived to have a positive impact on coping when providing information, objective care, emotional support, support resources, and follow-up care 3. TYhese practices encouraged confidence in their personal decision-making and gave women hope for the future
Sun et al., 2018 [92] **The experiences of fathers whose spouses are hospitalized for pregnancy termination due to fetal chromosome abnormality in Taiwan**	Taiwan	20 fathers	Semi-structured in-depth interviews	Qualitative Descriptive phenomenological approach Retrospective	The aims of this study were to explore and reveal the essence and structure of the experiences of Taiwanese fathers whose spouses are hospitalized for pregnancy termination due to fetal chromosome abnormality	Main themes: Four themes emerged: 1. A dismayed father: the unexpected process of terminating pregnancy 2. A hidden source of grief: neglected care 3. A stressful decision: difficulty handling the deceased offspring 4. A regretful father: inadequate treatment of the baby's remains
Zareba et al., 2018 [93] **Role of social and informational support while deciding on pregnancy termination for medical reasons**	Poland	150 women	Survey	Quantitative Retrospective Measures Used: An anonymous survey consisting of sixty questions to determine patient profile and forms of support expected from the society, family and professional medical personnel as well as to assess informational support provided Due to the subject of the study, the majority of variables were measured on a nominal scale. Therefore, descriptive statistics and descriptions were used. In the majority of cases, while examining the strength of the patients’ beliefs, especially on the 5-point Likert scale, Spearman’s rank correlation coefficient was used to measure the strength of the correlation	The aim of the paper was to determine the patients’ needs with regard to support provided by medical personnel and the healthcare system as well as to establish what forms of support the patients expect from their partner, family and people in their surroundings to experience their period of grief in the least traumatic way	Main findings: 1. Women do not take into consideration society’s opinion on pregnancy termination (95%) 2. The majority of the respondents think that financial support from the state is not sufficient to provide for sick children (81%) 3. Despite claiming to have a medium standard of life (75%), nearly half of the respondents (45%) say that they do not have the financial resources to take care of a sick child 4. The women have informed their partner (97%) and closest family members (82%) and a low percentage have informed friends (32%) 5. Nearly one third (31%) have not talked to the attending gynecologist about their decision

**Fig. 2:**
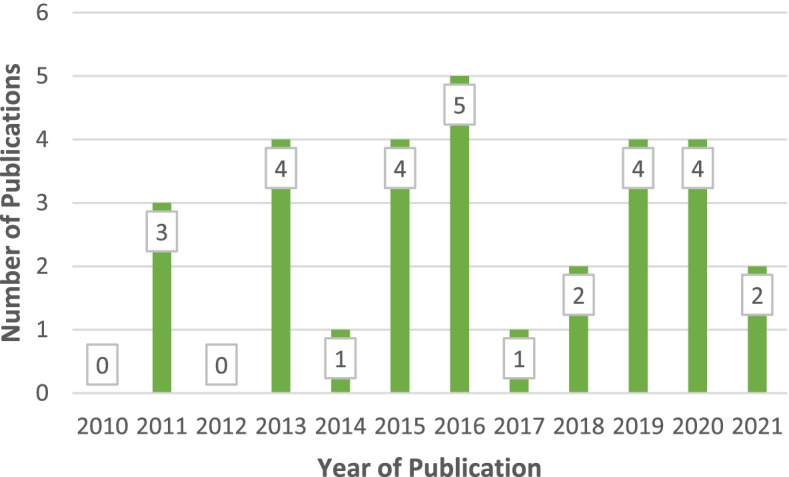
Year of publication

Of the six quantitative and mixed-method articles [68, 69, 72, 90, 91, 93], only one used a validated instrument [91]. The remainder were surveys and provided mainly descriptive quantitative data. The aim of the review was to explore healthcare experiences and needs, therefore the authors advocate using a narrative synthesis of quantitative data was appropriate and beneficial for this review. The articles were published between 2010 and 2021, with the majority (*n* = 22) published in 2015 or after (see Fig. 2). Sample size of included studies ranged from 7—361 participants, with an average of 48 participants per study. Eighteen studies included women only, five involved men only and five included both women and men. Collectively, this review is based on a total sample size of 1,227 women and 114 men.

The geographical spread of the studies includes United States of America (USA) (5), UK (4), Israel (3), Iran (2), Sweden (2), Australia (2), and one each from Spain, Thailand, The Netherlands, France, Finland, Canada, China, Taiwan and Poland. One study includes participants from USA and Canada [74]. 26 of the studies used a retrospective design and two a prospective one [89, 90].

### Quality appraisal of included studies

Due to the paucity of research and wide scope of this review no articles were excluded on the basis of quality. Two studies did not state whether ethical approval had been sought or obtained [70, 79], however, the majority of (29 out of 30) articles reported on their adherence to appropriate ethical standards. Two studies acknowledged potential conflicts of interest [72, 80], both relating to the author being involved with the charity that was used as a gatekeeper for recruitment. Two US studies reported offering participants a financial incentive for taking part [70, 73].

See supplementary file 1 for a summary of the quality of each study.

### Thematic synthesis findings

Five overarching themes emerged from the synthesis of findings: (1) Contextual impact on access to and perception of care, (2) Organisation of care, (3) Information to inform decision making, (4) Compassionate care, and (5) Partner experience. Table 3 summarises the themes and subthemes identified as part of this narrative synthesis.

**Table 3:** Mapping of themes to the included studies

Theme	Subtheme
**Contextual impact on access to and perception of care** The perceived impact and importance of broader contextual factors in parents’ access to and experience of care	Political-legal context Socio-cultural context Financial implications
**Organisation of care** The perceived impact of the administrative arrangements, service availability and physical environment on the perceived quality of the care experience	Service efficiency Workforce organisation Environment Aftercare
**Information to inform decision making** The perceived impact of responsive and respectful healthcare in helping parents feel empowered through this experience	Information Choice Decision Making
**Compassionate care** The perceived quality of the relationship a healthcare professional makes with parents and the impact on their satisfaction with their care experience	Empathy Experienced Staff Non-judgemental Staff
**Partner experience** The perceived impact of the health system and healthcare professionals in recognising and facilitating the involvement of partners	Invisible Parent Supporting Carers

### Contextual impact on access to and perception of care

Political, legal and cultural contexts are pertinent to both the healthcare system and experience of parents undergoing a TOPFA and can shape and impact healthcare service provision, directly and indirectly, including how it is accessed and experienced. Despite TOPFA being legal in 113 countries [19], 15 of these being represented in this review, there is evidence from the studies that parents’ experiences of TOPFA were impacted negatively by wider contextual factors including legislation, local procedures, professional practices and societal attitudes about TOP [73, 75, 76, 80, 84, 87, 93].

Challenges associated with legislation and policy included access to and availability of healthcare. These impacted on parents in a variety of ways, from having to travel or self-fund to access a TOPFA, to experiencing delays because of administrative ‘red-tape’, such as the need for committee approval. This led to delays in decision-making and referrals for the termination, leaving parents anxious about whether the outcome of the committee meeting would support their choice or not [73, 82–84, 87].

Funding of the TOP was a pertinent issue for participants from studies in the USA where private medical insurance added a layer of complexity and debate over whether the procedure was ‘elective’ or ‘medical’ with the answer determining whether funding would be provided or not, “a lot of insurance won’t cover elective procedures” [73]. This led to women taking action into their own hands with one woman, while awaiting insurance approval searching ways to ‘self-abort’, and another self-funding the procedure [73]. An Iranian study [76] reported financial issues experienced by parents, with some unable to access advanced screening or diagnostic tests and services such as genetic and psychological counselling due to high costs, “my doctor said, ‘you should have the genetic tests’, but the tests are expensive so my husband and I decided to wait” [76].

### Organisation of care

Well organised care, which was timely, efficient, and properly resourced was identified as a major contributor in parent’s satisfaction and was addressed in 18 of the 30 included articles [64, 65, 67, 68, 71–74, 76, 78, 81, 82, 84, 85, 87, 88, 90, 92]. Delays in appointments for further diagnostic tests and slow turnaround times for results were experienced as frustrating and increased parents’ anxiety [65, 71–74, 81, 82, 87, 93]. Any delay in accessing a TOPFA post diagnosis was perceived negatively, and in some cases seemed to impact on the choices available due to the gestational age of the fetus [71–74, 81, 82]. One UK study reported “increasing pressures around 13–14 weeks’ gestation, after which surgical terminations are harder to access in the NHS” [71]. Any obstacle or delay once the decision to have a TOPFA had been made increased parent’s stress with one participant comparing the wait for the procedure to “being on death row” [71].

Co-ordination and continuity of care were also highlighted as important elements of effective care [64, 65, 67, 68, 71–74, 76, 81, 82, 84, 85, 88, 92, 93]. In some instances, the handover of a woman’s medical history, notes and ongoing care between practitioners were inadequate, meaning those taking over the care were ill-informed, resulting in poor communication [64, 65, 71, 76, 80]. Failure to read case notes before seeing a patient led to upsetting experiences for some parents, “I had to tell her the baby had died. She hadn’t read the notes properly! I was furious and very distressed” [80]. For some, seeing a different doctor every time they attended the clinic resulted in them withholding their fears and concerns [65]. A call for continuity of care was specifically reported in four studies [64, 65, 71, 85].

Parents valued being cared for by experienced members of staff [64, 67, 69, 71, 73, 74, 76, 78, 79, 85, 89] and found it reassuring, “I felt I was being treated by experts” [71]. Conversely, being cared for by junior or inexperienced members of staff was found to be distressing and impacted on how confident and safe parents felt about their care, “normal midwives seemed not to know what to do. One told me that she had never delivered a stillborn baby. This was the last thing I needed to hear” [71].

In terms of setting, TOPFAs were carried out in hospital, clinic settings and in abortion clinics. Ten articles explored the significance to parents about being cared for in an appropriate environment [65, 67, 71, 74, 76, 82, 85, 86, 88, 92]. Negative experiences included; being surrounded by women with healthy pregnancies, being close to new mothers and crying newborns, physical indicators of celebration for a newborn, posters on walls of healthy newborns, and sharing waiting rooms with women terminating an unwanted pregnancy or women coming for a caesarean section.

There was no consensus about parents’ preferences on the most appropriate environment for their TOPFA. For some of those at an earlier gestation, gynaecological wards were preferred because they wished to avoid being in close proximity to newborns or to where other people were giving birth [65, 67, 71, 74, 85, 88, 92]. For others, being in a delivery unit validated their status as “a pregnant mum” [71]. For these women, the gynaecological ward felt inappropriate, separating them from ‘normal birth’, which compounded their sense of isolation [71, 85].

Aftercare was identified by some parents as a gap in the services available to them within the healthcare system, and for many was not routinely provided [64–66, 69–71, 74, 76, 77, 79, 80, 83, 87, 90, 91]. In the absence of aftercare being provided, women expected and wanted healthcare practitioners to signpost them to support organisations [64, 65, 69–71, 74, 79, 80, 85, 88, 90, 91]. However, it was reported that this often did not happen, and they had to assume personal responsibility to find, and in some cases privately fund, aftercare [64–66, 69–71, 74, 79, 80, 85]. For those who were signposted to or who accessed aftercare, they found it to be helpful and beneficial [64–68, 70, 71, 73, 74, 80, 84, 89], “the care was very good. [The] bereavement midwife [was] excellent and I saw her lots after [the TOPFA]” [71]. Parents expressed the view that the aftercare for those who had undergone TOPFA should be bespoke as it differs significantly from other types of perinatal losses and support groups for other infant losses were mostly considered to be unhelpful or inappropriate [64, 65, 71, 74, 80, 85, 87]. Preparation, information, and support before a potential future pregnancy was also raised as a concern and need for parents in two studies [75, 91].

### Information to inform decision making

Most articles (26 out of 30) addressed parents’ need for information and the impact a lack of information had on their experience [64–83, 85, 87, 89, 91–93]. While most parents acquired information themselves from a range of sources, such as “healthcare professionals, books, the internet and from individuals who have been in similar situations” [74], clear and unbiased information provided by healthcare professionals was greatly valued. When parents were given relevant and timely information, particularly about the anomaly and healthcare procedures [64–69, 71, 72, 74, 75, 77, 80–82, 85, 87, 89, 91–93], it reduced their fears and worries, helped them understand their choices, and feel more empowered [64–67, 71, 73–75, 79, 80, 82, 85, 87, 92, 93]. Parents who felt ill-informed at any stage in the process felt less well-prepared physically and psychologically about what to expect and, for some, their experience was more traumatic [64–67, 71–74, 76, 77, 79, 82, 83, 85, 92].

Some studies reported parents’ frustration at trying to find information, while others expressed frustration about inconsistent and conflicting information [64, 65, 71, 73, 74, 79, 82], “every time I phoned asking this very same question, I received different answers. Very exhausting” [64]. This also resulted in dissatisfaction and suspicion about the quality of any information provided, with some parents feeling that medical staff were withholding information, “we deserved to know what they knew” [73]. Being given inappropriate information was distressing, “[it was unhelpful] being handed a leaflet about dealing with a miscarriage almost immediately afterwards when I was clearly dealing with an awful decision which was NOT a miscarriage” [71].

Several studies reported how parents rated the way information was communicated to them [64–68, 71, 74, 79–82, 84, 85, 87, 91]. Parents generally felt that written information without the opportunity to discuss it and ask questions was not helpful, “they just gave me a piece of paper, but that’s not the same as actually talking it through with someone in person” [79]. There was no consensus about the use of language by healthcare providers, with some parents critical of the use of medicalised terminology [65, 71, 74, 79, 82, 84], for example, referring to the baby as a “product of conception” [71] while others were critical of those who referred to “the fetus as a baby” [71].

For many study participants the point of diagnosis was pivotal, marking the beginning of a different journey where decisions about the future of the pregnancy had to be made [65–67, 70, 73–75, 78, 81, 87–89]. This encounter was described as difficult and emotional [65–67, 70, 71, 74, 75, 78, 79, 81, 82, 89, 90]. Overall, being given choices was seen as positive and empowering [64, 65, 69–74, 79, 81, 82, 87, 89, 90], not just the choice to end the pregnancy but other choices throughout the process, such as seeing and holding baby and what to do with baby’s remains. However, making decisions was described as difficult and involved conflicting feelings. Pain relief, for example, was identified as an important decision by some, “I was given pain relief whenever I needed it” [79], both the choice to have it and the choice of what pain relief medication they had [64–66, 79, 80, 82, 85]. The studies that reported on pain relief suggested that most women wanted to disconnect from the process and avoid unnecessary suffering, and the use of analgesia or sedation helped this, “I was offered some pethidine for the pain, and although I wasn’t in pain I accepted it, it numbed my brain and helped me sleep” [80]. Being overwhelmed by the decisions to be made was reported in several studies [65–67, 69–72, 74, 77, 81, 89, 92]. A study which focused entirely on whether women were given a choice of method of TOPFA [72] found that only 14% of the sample were, with this number falling to 8% after 14 weeks gestation. In this study, almost half of women (47.6%) when asked had they had the procedure which best suited them were ambivalent, disagreed or strongly disagreed. Women who had a surgical procedure were more likely to report positively they had the procedure which suited them compared to women who had a medical TOPFA (73.1% vs 47.3%).

### Compassionate care

Healthcare providers’ capacity to provide compassionate and empathetic care presented as potentially the most influential element in how parents perceived whether their experience was positive or negative. Compassionate care was explored in 21 out of the 30 articles [64–71, 74–76, 78–82, 84, 85, 87, 91, 93]. Women were most satisfied with providers when they responded to their communication and emotional needs [64–69, 71, 74, 75, 79–82, 84, 85, 91, 93], as highlighted in a UK study, “the consultant also held my hand tight [...] this warmth from the staff I will always remember” [80]. A perceived lack of empathy and kindness had lasting impact after the experience, as highlighted by a study in Canada and USA, "the supervisor nurse was kind of brusque and not very friendly and I unfortunately remember that quite clearly" [74].

Healthcare professionals who were perceived to be non-judgmental and who showed kindness and support for parents were greatly valued, “one of the kindest people during the whole process was the anaesthesiologist who held my hand […] and said he understood I was making the right choice” [74]. The importance of non-judgmental staff was highlighted in eleven studies [65, 70, 71, 73–75, 81, 82, 85, 87, 93]. Parents used words such as ‘shame’ and ‘guilt’ to describe how they felt about having a TOPFA, and perceived judgement or stigmatisation from healthcare professionals was experienced negatively, “I felt she [midwife] made me feel unworthy for my decision” [71].

Parents appreciated practitioners who cared for their baby with tenderness [71, 74, 79–81, 85–87, 92], “the care and attention the midwife on duty showed to our son […] talking to him as she washed and dressed him” [71] and were distressed when this was not the case. While some parents were ambivalent about spending time with their baby, the most positive experiences were reported by those parents who were encouraged and helped to create memories as well as were given as much time as they wanted with their baby [65–67, 69, 74, 78–81, 84–87, 92]. The facilitation of this by health practitioners was appreciated by parents, “we were allowed to look at him in peace. He was only taken away when we were ready” [79].

Compassionate care was manifested in the ability of the healthcare system and willingness of staff to tailor care responsively to each parent’s circumstances [64, 67, 71, 78]. This included fast-tracking people for basic procedures, such blood samples, so they did not have to face additional waiting times [71, 88]. There was also appreciation when staff ensured women spent little time in open public waiting areas or escorted them quickly and discreetly to a private space [67, 71, 74, 85]. Satisfaction was also expressed when staff helped make other healthcare professionals aware of their loss to avoid inappropriate comments or questions by staff, “they put a white flower on my door to let them [the staff] know that I was not leaving with a baby” [74].

### Partner experience

Several studies reported that partners felt excluded or ignored by healthcare staff, and highlighted how the healthcare system in general, and organisational issues in particular, were not designed to cater for them or enable them to support the pregnant woman [70, 76, 78, 83, 85, 87, 92]. Partners often felt excluded, ill-prepared and unwelcome, with a partner in one study stating “it does feel a bit like they forget the father sometimes you know. It was like the bed in the hospital and there was no bed for me. You know, not even a blanket, and [the midwife] said there wasn’t enough pillows [for me to have one]” [85]. Some rationalised their exclusion and/or apparent invisibility because “pregnancy is a woman’s issue […] it’s a no man’s land” [83]. Additional to the hospital environment, fathers also reported a lack of specific aftercare and support targeted towards men, resulting in them feeling uncertain and conflicted between their roles as grieving father and supporting their partner. A father said, “they sent two counsellors in to speak to us together […] unless I spoke up and said something […] she looked at [wife]. I’m sitting there, and all I’m hearing is: I have to look after her. I have to support her. I have to make sure she’s okay. I have to be strong enough to bear the weight of my own grief, as well as support the weight of my wife’s grief” [87].

In some studies, partners identified themselves as the main or only support for their pregnant partner [69, 70, 78, 83, 87, 92]. Women also recognised the central support role their partners played [65, 67, 73, 80, 85, 93]. While partners wanted to care and support their pregnant partner throughout the TOPFA process, this was more difficult for some when there was “little professional assistance, empathy or caring” [83]. One woman reported, “my husband actually delivered the baby ‘cause there was nobody there” [85]. In another case, the partner reported, “she was bleeding […] and they [professionals] did nothing! I felt abandoned […] I took care of her” [83]. Partners, in these circumstances, felt “forced to take the situation in [their] own hands” [83]. When professionals supported women and their partners, the experiences of partners were much more positive, “all the people who assisted us […] were very competent and very nice and that greatly helped” [78].

## Discussion

This systematic review synthesises findings from the international literature on the healthcare experiences and needs of parents who undergo a termination of pregnancy following an antenatal diagnosis of a fetal anomaly. As noted above, others have reviewed the evidence concerning this phenomenon from other timeframes and perspectives [33–39, 44]. To our knowledge, this is the first review exploring the holistic TOPFA healthcare experience, from diagnosis to aftercare from both parents’ perspectives. Using the process of thematic analysis this review enhances the knowledge and understanding of the TOPFA healthcare experience and needs of parents and identifies factors that may impact their experience.

Five over-arching themes containing 15 sub-themes, generated from 30 articles (28 studies), were interpreted in the thematic synthesis. The overarching themes related to: (1) contextual impact on access to and perception of care, (2) organisation of care, (3) information to inform decision making, (4) compassionate care, and (5) partner experience. A key message, evident across included studies, was that a one-size-fits-all approach to care is not acceptable. Instead, the findings suggested that well-organised and compassionate, high quality care adapted to individual needs was optimal.

### The importance of compassionate care

The findings relating to the need for compassionate care are congruent with other literature regarding TOPFA, which highlights the importance and impact of compassionate relationships with healthcare professionals for parents in this situation [44, 50, 80]. While these findings are not dissimilar to the experiences of every pregnant woman [94], the circumstances and the outcome of a TOPFA makes this different [95–97]. Women and their partners want and need staff to connect with them as grieving parents and to acknowledge and respond to the loss of their baby [98–100].

### Informed decision-making

The findings relating to informed decision-making reflect those of other studies [101, 102] and are in keeping with the shift from a paternalistic model of care to an inclusive and empowering person-centred model where care is responsive and individualised to a persons’ needs [103–110]. The decision to proceed with a TOPFA is acknowledged by accounts in this review as a conflicted and difficult choice [65, 67, 77, 81, 92]. In line with existing literature investigating decision-making in relation to TOPFA [111, 112], the included studies suggest parents valued and appreciated health professionals who provided information, support and validation of the decision as opposed to a recommendation for a TOPFA. The provision of timely and relevant information by staff is one practical step that could help empower parents to understand what choices they have and give them a greater sense of autonomy in a situation over which they feel they have little control [113–115]. Further work is needed from both a parent and health professional perspective to explore the complexities of decision making regarding TOPFA.

### Supporting partners

Research in pediatric and neonatal bereavement has seen an increasing focus on meeting the needs of the father following the loss of a child [116–119]. The findings in this review suggest that, with a few exceptions, partners had a negative experience and felt excluded and ignored, and needed more support both as a grieving parent and in their role as carer to the pregnant woman [70, 78, 83, 85, 120]. Awareness raising and training about the need to be inclusive of a woman’s primary support network (with her permission), and the partner’s needs for support both as a grieving parent and as a carer could enhance their experience in the future [121–123].

### Practice implications

The findings of this review suggest that healthcare professionals would benefit from support and training to meet the needs of parents undergoing TOPFA [124–126]. Of particular importance to the participants of the included studies was effective communication between patients and healthcare practitioners. The findings suggest that a regular and sustained focus on communication skills throughout practitioners’ careers would be beneficial and could support a better-quality service [104]. Examples of how this could be achieved include, patient feedback, staff appraisal, refresher training or revalidation [127–133].

Relatedly, the findings suggest that continuity of care could potentially support better experiences for parents following the diagnosis of a fetal anomaly [64, 65, 70, 85]. Over recent years the evidence supporting continuity of care in maternity contexts is well documented in the literature [134–136] and is increasingly seen in government policy and strategies across healthcare disciplines [137–140]. Further, there has been an increasing focus on baby loss in maternity care and a recognition of the need for specialist bereavement midwives and services [141–143]. These developments, although still in their infancy in terms of service development, have the potential to benefit all parents who lose a baby, including those who have experienced TOPFA. Given the potential impacts that adequately trained and dedicated staff can have on mental health outcomes [16, 33–39], resources should be focused on maintaining and developing these posts and services.

This review supported the findings of other studies that there was no consensus about the preferred setting for a TOPFA procedure [71, 74, 82, 85, 88]. This implies that, where feasible, women should be given the choice of setting [104]. Where it is not feasible and women have to be treated in a maternity ward, this needs to be managed sensitively in ways that minimise further distress for those who have lost a baby [71, 85].

### Policy implications

Changes in practice often require prerequisite changes in policy and care pathway guidelines. While recognising there are some national policies and guidelines regarding TOPFA [144–146], many parents in this review reported a disjointed care pathway that did not fully meet their needs [78, 81, 84]. It is important for health professionals to have evidence-based and structured pathways that ensure parents’ individual care needs are addressed [147]. As with any guidance or care pathway, scope for professional judgement would need to be included to allow for a person-centred care approach to meet the specific needs and preferences of each woman and her partner and the unique circumstances of their case [148]. Findings in this review, supported by other literature, advocate pathways should consider short- and longer-term needs, including, for example, choices regarding clinical procedures, aftercare, and post-TOPFA psychological support [39, 44, 77, 149, 150].

The findings confirm that the provision of services and care in respect of TOPFA varies between and within countries due to a wide range of legal, socio-economic and cultural reasons [73, 82, 120]. There was, however, a common theme that impacted on individual’s experiences, that is the perceived judgement surrounding termination of pregnancy, which continues to be a divisive issue within most societies [151–154]. In countries where TOPFA is legal, awareness raising, education and information about ethical practice, human rights and conscientious objection could lead to greater understanding and help reduce potential stigma surrounding TOPFA [155, 156]. Anonymised stories of parents who have experienced TOPFA and the experiences of healthcare professionals could also be used to raise awareness of the issues and address barriers to care related to perceived stigma or judgement [157, 158].

### Strengths and limitations

This systematic literature review and thematic synthesis enhances existing literature and to our knowledge, it is the first mixed-methods systematic examination and narrative synthesis of the healthcare experiences of both parents regarding TOPFA. A strength of the review is the utilisation of guidelines and best practice procedures for carrying out a systematic review, such as protocol publication to ensure a robust and transparent process. The inductive approach to data analysis, as well as the rigorous and comprehensive search strategy for relevant literature and the evident auditable trail clearly demonstrated the journey from primary studies to interpretations. Independent analysis by two members of the review team enhances credibility.

This review has several limitations. Firstly, it only includes articles written in English, possibly omitting significant or insightful studies. There is a small evidence base per country context, and although not the purpose of this review, subsequently means results may not be directly transferrable to different settings, contexts or samples. There is an over-representation from high-income countries [159], drawing attention to possible differences in antenatal care pathways and availability and access of screening and diagnostic services in different countries. Studies in the UK and USA were also over-represented, as well as the majority of participants in studies being from a white ethnic background, middle-class and well educated. This possibility of cultural bias has been noted in other studies exploring this phenomenon [39, 160, 161]. All studies used convenience, purposive or snowball sampling to recruit participants. While ethically appropriate and justified in relation to the nature of the research, it is important to note that results may not be representative of all parents who experience TOPFA. For a review focusing on both parents’ experiences, those of the partner made up less than 10% of the total sample the findings are based upon, highlighting the dearth of, and need for, more research involving partners in the field of maternity and perinatal care and loss.

### Opportunities for future research

In-depth primary research with both parents and health professionals would be beneficial and could help inform and improve service delivery and parents’ experiences. Primary qualitative research exploring other family members experience may also be beneficial as literature alludes to the main support for parents being firstly their respective partner followed by their family network. There is also a need to develop and evaluate interventions with this group aimed at improving their healthcare experience and health outcomes.

## Conclusions

The findings from this review highlight the individual nature of people’s experiences and responses to the healthcare they received in respect of TOPFA. They also emphasise the resulting need for an individualised approach to healthcare. Importantly, the findings indicate a degree of consensus about how appropriately trained health professionals, compassionate and person-centred care, good information and communication, and a thoughtful and integrated care pathway, can help make parents feel supported and cared for through what is an emotionally traumatic experience.

## Supplementary information

**Additional file 1.** Quality appraisal of included studies.

## Data Availability

The datasets used and/or analysed during the current study available from the corresponding author on reasonable request.
