# Supplementary material for: Termination of pregnancy for fetal anomaly: a systematic review of the healthcare experiences and needs of parents
Source: BMC Pregnancy Childbirth. 2022 May 26;22:441. doi: 10.1186/s12884-022-04770-4 (PMC9137204; doi:10.1186/s12884-022-04770-4)
Supplement: Supplementary file 1 — Additional file 1. Quality appraisal of included studies. [file 12884_2022_4770_MOESM1_ESM.pdf]

Supplementary File 1: Quality Appraisal of Included Studies

STUDY	Screening Questions **ALL** studies		QUALITATIVE STUDIES					QUANTITATIVE DESCRIPTIVE STUDIES					MIXED METHODS STUDIES					Comments
	Are there clear research questions?	Do the collected data allow to address the research questions?	1. Is the qualitative approach appropriate to answer the research question?	2. Are the qualitative data collection methods adequate to address the research question?	3. Are the findings adequately derived from the data?	4. Is the interpretation of results sufficiently substantiated by data?	5. Is there coherence between qualitative data sources, collection, analysis and interpretation?	1. Is the sampling strategy relevant to address the research question?	2. Is the sample representative of the target population?	3. Are the measurements appropriate?	4. Is the risk of nonresponse bias low?	5. Is the statistical analysis appropriate to answer the research question?	1. Is there an adequate rationale for using a mixed methods design to address the research question?	2. Are the different components of the study effectively integrated to answer the research question?	3. Are the outputs of the integration of qualitative and quantitative components adequately interpreted?	4. Are divergences and inconsistencies between quantitative and qualitative results adequately addressed?	5. Do the different components of the study adhere to the quality criteria of each tradition of the methods involved?	Comments
64	Y	Y	Y	Y	Y	Y	Y											TOPFA full sample
65	Y	Y	Y	Y	Y	Y	Y											TOPFA full sample
66	Y	Y	Y	Y	Y	Y	Y											TOPFA full sample
67	Y	Y	Y	Y	Y	Y	Y											TOPFA part of sample<br>PhD Thesis
68	Y	Y						C	C	C	C	C						TOPFA full sample<br>Small, single hospital study. The lead author works in the 'Center for Spirituality, Theology and Health' at the Medial Center, so a potential bias was queried, although none declared. There are grammatical errors in the document, limited detail on methodology and a financial incentive for taking part in study was offered to participants ($40 gift card).
69	Y	Y						Y	Y	Y	C	Y						TOPFA full sample<br>47% non-response rate. Not discussed but assumed due to nature of study not surprising. No indication that

																		participants greatly differed from those who did not respond.
70	Y	Y	Y	Y	Y	Y	Y											TOPFA full sample<br>$25 gift card incentive offered.<br>Masters Thesis<br>Did not state whether ethical approval had been sought or obtained
71	Y	Y	Y	Y	Y	Y	Y											TOPFA full sample<br>TOPFAs were performed between 1969 and 2012. Most (75.7%, n=265) were performed after 2006
72	Y	Y						Y	Y	Y	Y	Y						TOPFA full sample<br>Conflict of interest declared - the lead author is the Director of ARC (recruitment avenue)
73	Y	Y	Y	Y	Y	Y	Y											TOPFA full sample<br>Participants were compensated with a $40 gift card
74	Y	Y	Y	Y	Y	Y	Y											TOPFA full sample<br>PhD Thesis
75	Y	Y	Y	Y	Y	Y	Y											TOPFA part of sample
76	Y	Y	Y	Y	Y	Y	Y											TOPFA full sample
77	Y	Y	Y	Y	Y	Y	Y											TOPFA full sample
78	Y	Y	Y	Y	Y	Y	Y											TOPFA full sample
79	Y	Y	Y	Y	Y	Y	Y											TOPFA full sample<br>Did not state whether ethical approval had been sought or obtained
80	Y	Y	Y	Y	Y	Y	Y											TOPFA full sample<br>The authors declared the following potential conflict of interest with respect to the research, authorship, and/or publication of this article: Caroline Lafarge is a member of the volunteers network of Antenatal Results and Choices (recruitment avenue).
81	Y	Y	Y	Y	Y	Y	Y											TOPFA full sample<br>The studies used different methods of data collection and therefore elicited different types of data. The data collected through online narratives (Study 1) generated reflective accounts and provided insights into the meaning women attributed to their experience. Nevertheless, the data did not offer the same level of detail as the data

																		collected through in-depth interviews (Study 2). By contrast, although inter-viewing participants enabled the researchers to collect more granular data (through probing), the topic guide was broader and less focused on the meaning of women's experience. However, both studies generated rich data and both achieved data saturation.
82	Y	Y	Y	Y	Y	Y	Y											TOPFA full sample
83	Y	Y	Y	Y	Y	Y	Y											TOPFA full sample
84	Y	Y	Y	Y	Y	Y	Y											TOPFA full sample
85	Y	Y	Y	Y	Y	Y	Y											TOPFA full sample
86	Y	Y	Y	Y	Y	Y	Y											TOPFA full sample
87	Y	Y	Y	Y	Y	Y	Y											TOPFA full sample<br>Part of a larger study. Only TOPFA reported in this paper.
88	Y	Y	Y	Y	Y	Y	Y											TOPFA full sample
89	Y	Y	Y	Y	Y	Y	Y											TOPFA full sample
90	Y	Y						Y	Y	C	C	C						TOPFA full sample<br>No details on how survey designed. Follow-up surveys were not linked to the initial or previous survey due to restrictions placed by the Institutional Review Board.<br>T1 - 51; T2 - 6; T3 - 4. Very low follow up rate - authors acknowledge this - Given this low response rate, analysis of the follow-up surveys was limited.
91	Y	Y											Y	Y	Y	Y	Y	TOPFA full sample
92	Y	Y	Y	Y	Y	Y	Y											TOPFA full sample
93	Y	Y						C	C	C	C	C						TOPFA full sample<br>Article provided limited detail on methodology. No inclusion or exclusion criteria stated. No details of demographics given.<br><br>The main concern about this study was in relation to the recruitment. It was stated patients were asked to take part on admission to hospital for treatment by one of the clinical care team, prior to TOPFA procedure. The study reported a high response rate, however the ethicality of this practice was

																		questioned as it raised concerns for two reasons. Firstly, recruiting prior to the provision of healthcare may have influenced women taking part due to a fear of treatment being withheld or other impact on their care, especially in the context of Poland, where TOP has recently been restricted. Secondly, recruitment at this time gives limited time for reflection or space to consider participation.

Y – Yes

N – No

C – Can't Tell
